# Psychometric Properties of Eating Behaviour Instruments for Older Adults: A Systematic Review

**DOI:** 10.1111/jhn.70127

**Published:** 2025-09-23

**Authors:** Giulia de Pádua Constâncio, Gisele Teixeira de Souza Silva, Maria Fernanda Ferrari Bahia, Marcela Rodrigues de Castro

**Affiliations:** ^1^ Federal University of Bahia Salvador Bahia Brazil

**Keywords:** eating behaviour, older adults, psychometric

## Abstract

**Introduction:**

Although eating behaviour in children, adolescents and adults has been linked to changes in body mass, eating disorders and non‐communicable diseases, there is still uncertainty about how this behaviour has been measured in populations aged 60 and over, who are often exposed to age‐related physiological and cognitive changes, increased malnutrition risk and underrepresented in psychometric research.

**Aim:**

To analyse how eating behaviour has been investigated in people aged 60 and over.

**Methods:**

Following the Preferred Reporting Items for Systematic Reviews and Meta‐Analyses (PRISMA) guidelines and the Consensus‐based Standards for the Selection of Health Measurement Instruments (COSMIN), three independent researchers selected articles from four databases from 2000 to the present. Inclusion criteria: (1) Studies on adaptation, development, or evaluation of psychometric properties of eating behaviour instruments; (2) Studies with participants aged ≥ 60 years, or with a significant proportion/stratified results for this group; (3) Publications in English, Spanish, or Portuguese; and (4) Articles published from 2000 to 2024. Exclusion criteria: (1) Studies on food consumption; (2) Instruments for diagnosing eating disorders; and (3) Non‐original articles.

**Results:**

Eight instruments were identified for assessing eating behaviour in older adults, with the majority developed in Europe. Methodological shortcomings were present across all instruments, particularly in the areas of development, content validity, internal validity and consistency.

**Conclusions:**

All instruments exhibit questionable methodological robustness, highlighting the need for more rigorous and culturally sensitive approaches to the development and adaptation of tools for this population.

## Summary

- Few instruments have been developed or adapted to assess eating behaviour in older adults.
- Available tools require stronger psychometric rigour and culturally sensitive adaptation for this population.
- Eight instruments were identified, including versions of the TFEQ and YFAS, along with the DEBQ, MEBS, FABB and MES.

## 1 Introduction

Healthy active ageing has been advocated by the world's leading health agencies [1]. Nutritional guidance, which involves identifying dietary intake and using behavioural therapy to better monitor eating behaviours (EBs), is recognised as an essential strategy [2]. EB is characterised as a set of cognitive processes that regulate actions related to eating [3, 4].

In people over 60 years old, factors such as life experiences, valuing nutrition, interest in health, sociability, focusing on one's own EB without discussing it with others or commenting on others' behaviour, can lead to positive EBs, such as mindful eating, respecting hunger signals, making healthy food choices, among others [5, 6]. Conversely, living alone, food prices, lack of social support (formal or informal) for shopping and meal preparation, and lack of quality information can lead to negative EBs, such as uncontrolled eating, poor food choices, ignoring satiety signals, or restricting food intake with the intention of losing weight [5, 6].

To assess and monitor the aforementioned EBs, appropriate evaluation instruments that have undergone psychometric validation are necessary [7]. This process is essential for research and occurs in stages: evidence based on test content, evidence based on relationships with other variables, and evidence based on internal structure [7].

Globally, various instruments have been developed to assess different EBs across diverse populations. The Restraint Scale (RS) [8] measures cognitive restraint, although later studies demonstrated that the instrument also assesses weight fluctuation and the effects of social desirability, features that can distort RS scores in some groups [9, 10, 11]. The Three‐Factor Eating Questionnaire (TFEQ) [10] was developed to measure multiple aspects of EBs, such as cognitive restraint, disinhibition [10] and hunger (feelings of hunger and associated behaviours) [10]. The TFEQ has been translated and adapted for various populations [12, 13, 14, 15]. In 1986, the Dutch Eating Behaviour Questionnaire (DEBQ) [16] was developed, which measures external eating, cognitive restraint and emotional eating. It has also been validated for various populations [17, 18, 19].

However, it remains unclear which instruments have been used to evaluate the older population groups on a global scale or how EBs are being measured. This lack of clarity undermines the ability to conduct accurate assessments and prescribe assertive interventions. Therefore, this systematic review aimed to identify available instruments that have demonstrated validity to measure EBs among people aged 60 and over.

## 2 Methods

The recommendations of the Preferred Reporting Items for Systematic Reviews and Meta‐Analyses (PRISMA) [20] were followed, and the article was registered at the International Prospective Register of Systematic Reviews (PROSPERO), protocol number CRD42024540845.

To define the descriptors to be used in the search, tests were carried out using terms available in the Health Sciences Descriptors (DeCS) related to the topic, such as feeding behaviour, elderly, aged, instruments, measure, EB and psychometrics. The combination that yielded the highest number of articles on instruments measuring the EB of older adults was in Portuguese, ‘comportamento alimentar’ and ‘psicometria’; in Spanish, ‘Conducta Alimentaria’ and ‘Psicometría’; and in English, ‘Eating behaviour’ and ‘Psychometrics’.

Boolean operators (AND and OR) were defined according to the specifications of each database—Scopus, ScienceDirect, PsycINFO and Medline. Across all databases, ‘OR’ was used for searches in Portuguese and Spanish, while ‘AND’ was used for searches in English. The research occurred during the period from 6 July 2023 to 25 July 2023 and was updated on 5 October 2024.

The inclusion criteria were as follows: (1) studies involving the adaptation, development, or evaluation of the psychometric properties of instruments that assess EB; (2) studies that assessed the psychometric properties of EB instruments in populations with a mean age of 60 years or older, or studies that had a significant proportion of individuals aged 60 or above, or presented results stratified to include a group of participants aged 60 or above; (3) studies published in English, Spanish, or Portuguese; and (4) articles published from the year 2000 onwards. The exclusion criteria were: (1) articles focused on food consumption; (2) articles involving the adaptation, development, or psychometric evaluation of instruments designed to diagnose eating disorders; and (3) non‐original articles. The search period for articles was defined between 2000 and 2024, as this marks the time when the construct of EB began to be more thoroughly studied [3].

**Figure 1:**
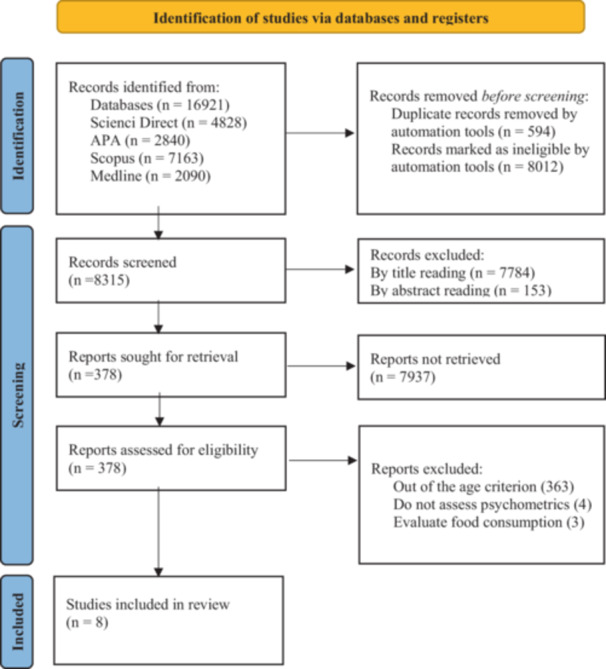
Flowchart of the article selection process.

The database searches were independently performed by three researchers. The article selection process is detailed in Figure 1. For the exclusion of articles based on title, abstract, and full‐text reading, the Rayyan software was used with the ‘blind on/off’ function activated to ensure impartiality during the selection process. Each researcher independently voted on whether to select the article according to the exclusion criteria. At the end of each stage, the function was deactivated, and conflicts were discussed in meetings with an advisor.

The data extracted from the articles included in the review were: author, journal, year of publication, location of the authors' university, location of the sample, instrument name, instrument dimensions, number of instrument items, response format, presentation format, application format, conceptual model followed (‘Trinitarian Validity Framework’ [21] or ‘Standards’ [7]), evidence based on content, evidence based on internal structure, and evidence based on the relationship with other variables [7]. When articles were not openly accessible or lacked specific information, access was requested from the authors via email or the ResearchGate platform. If the information was still unavailable, it was assumed that the author did not perform the step in question.

To ensure consistency and reliability in the data extraction process, the researchers involved in the article selection phase independently conducted the data extraction. Any disagreements were resolved by consensus or, if necessary, through consultation with the advisor, who ensured methodological and scientific rigour in the review. Data collection and organisation were carried out in an Excel spreadsheet following a standardised format for each criterion, facilitating the comparison and synthesis of the collected information.

To assess the risk of bias, the COSMIN checklist was used [22]. This framework is a widely recognised standard for evaluating the methodological quality of studies investigating the psychometric properties of health measurement instruments [22].

The primary reviewer along with one of the supervising experts in applied psychometrics, independently applied the COSMIN checklist using Excel. Subsequently, a synthesis of the results was conducted collaboratively. By applying these criteria, it was possible to objectively evaluate the quality of the studies included in the review, ensuring a detailed analysis of any potential bias present in the analysed studies, thereby contributing to the reliability and integrity of the presented results.

Each included study is evaluated according to specific boxes that represent different measurement properties, such as content validity, structural validity, internal consistency, reliability, measurement error, criterion validity, hypothesis testing, responsiveness and cross‐cultural validity [22]. Each box contains a set of items that must be rated based on the methodological quality of the study using a 4‐point scale: *very good*, *adequate*, *doubtful*, *inadequate or not applicable* [22]. The lowest rating across the items within a given box determines the overall score for that measurement property. The completed checklist allows for a standardised and transparent evaluation of the quality of the psychometric evidence provided by each study, supporting the assessment of risk of bias across all included instruments [22].

## 3 Results

Of the 16,921 articles identified across databases, 8606 were excluded after specific filters (Figure 1). In initial screening, 7937 records were excluded by title and abstract (Figure 1). For example, in title screening, one reviewer excluded 7801 publications, another 7426, and a third 7770. In abstract screening, one reviewer excluded 150 articles, another 152 and a third 160. These independent assessments preceded the consensus totals in Figure 1. In full‐text review, 370 articles were excluded for not meeting the inclusion criteria (Figure 1). This exclusion also stemmed from independent assessments and subsequent consensus; for instance, two researchers each excluded 370 articles, while the third excluded 375. Eight articles were included in this review. Following database updates, no new articles were added (Figure 1).

Table 1 presents: title, author, year and journal; article objective; country of the universities involved and the sample location; sample characteristics; instrument (dimensions/response, scale/application, format/development and model); and psychometric properties. Results are based on the percentage distribution of findings from selected articles (Table 2).

**Table 1:** Data extracted from the articles included in the review.

N	Title, author, year and journal	Objective of the article, country of the universities involved and the sample and sample characteristics	Instrument (Dimensions/Response Scale/Application Format/Development Model)	Psychometric properties
1	Psychometric Evaluation of the Three‐Factor Eating Questionnaire‐R18 in Elderly Finnish Men at Increased Risk of Type 2 Diabetes Malkki‐Keinänen et al. (2022) Nutrition and Health	To investigate the psychometric properties of the Three‐Factor Eating Questionnaire‐R18 (TFEQ‐R18) in elderly men at elevated risk of type 2 diabetes. Finland 420 men aged between 50 and 75 years (66 ± 6).	TFEQ‐R18, a multidimensional instrument that evaluates eating behaviour. It consists of 18 items subdivided into 3 dimensions: restrictive eating (6 items), uncontrolled eating (9 items) and emotional eating (3 items). Likert‐type response scale ranging from 1 to 4 points (1 = ‘definitely false’ to 4 = ‘definitely true’), with reverse scoring applied to Items 1–13. After summing the scores, a 0%–100% rescaling is performed using the formula: [(raw score − lowest possible raw score)/possible raw score range] × 100. A higher percentage indicates a greater tendency to exhibit the characteristic measured by the factor. Self‐Report: Participants must complete the instrument either online or in print and send it by mail. Tripartite Model.	*Evidence Based on Content*: The translated version by Karlsson et al. [11]—TFEQ‐R18 was used for the analyses. No analysis was conducted for Evidence Based on Content. *Evidence Based on Internal Structure*: Analysed through confirmatory factor analysis (CFA), reliability, composite reliability, average variance extracted and the adequacy of the tested models—relative chi‐square, incremental and fit indices (comparative fit index [CFI]) and two absolute fit indices—Root mean square error of approximation (RMSEA) and Standardised root mean square residual. *Evidence Based on Relations with Other Variables (Convergent and Discriminant)*: Average Variance Extracted and Composite Reliability. The model's adequacy included these indices. The distinguishability of the questionnaire factors was examined using method bias tests, subfactor correlation and multicollinearity. Correlations between TFEQ and age, BMI, and circumference were performed.
2	Development of the Short Version of the Modified Yale Food Addiction Scale, Version 2.0, in a Representative Sample of the Czech Population Pipová et al. (2020) Journal of Eating Disorders	Create a Czech Version of the Modified Yale Food Addiction Scale 2.0 (mYFAS 2.0) and Test Its Psychometric Properties in a Nonclinical Sample of the Czech Population. Explore the connection between food addiction, attachment style and sociodemographic characteristics. Czech Republic Sample: 1841 individuals (943 women) aged 15–70 years (28% over 60 years old).	The mYFAS 2.0 is a unidimensional instrument designed to assess food addiction based on individual dependency criteria as established by the DSM‐5 or clinical significance. It consists of 13 items. Response Scale: An 8‐point Likert scale ranging from 1 ‘never’ to 8 ‘every day’. Hetero‐Report Administration: The administrator presents the instrument online or in print and assists with completion. Tripartite Model.	*Evidence Based on Content*: The mYFAS 2.0 was translated from English to Czech. However, no data are provided regarding Language Clarity, Theoretical Relevance and Practical Pertinence. *Evidence Based on Internal Structure*: Analysed using CFA, model fit indices and reliability measures to assess the adequacy of the tested models. *Evidence Based on Relations with Other Variables (Convergent and Discriminant)*: Regression analysis was conducted to compare the mean values of the Czech mYFAS 2.0 across various sociodemographic groups (gender, age, living arrangements, marital status, education level, household income and smoking status) and different attachment styles (Relationship Questionnaire [RQ]—describes four relationship styles in four concise paragraphs: secure, avoidant, preoccupied and fearful).
3	Dutch Eating Behaviour Questionnaire (DEBQ): Assessment of Eating Behavior in an Aging French Population Bailly et al. (2012) Appetite	Test the factorial validity and internal consistency of the short version of the Dutch Eating Behavior Questionnaire (DEBQ) in an elderly population. Assess the factorial validity and the similarity of the factorial structure between men and women, between elderly and older individuals, and between those who were overweight or not (BMI status). France 262 individuals (178 women) aged 65–90 years (73.49 ± 5.46).	DEBQ is a multidimensional instrument capable of measuring eating motivations. It consists of 16 items assessing 3 dimensions: restrained eating (5 items), external eating (5 items) and emotional eating (6 items). Response scale: 5‐point Likert scale (1 = ‘never’ to 5 = ‘very frequently’). Not reported. Tripartite Model.	*Evidence Based on Content*: The DEBQ was adapted from the already translated French version [47]. Evidence Based on Content included a pilot study with the target population. A team of experts in eating behaviour and gerontology examined the factor loadings of the 33 items that make up the DEBQ, then assessed the relevance of the items in relation to ageing, and after adaptations, conducted a pilot study. *Evidence Based on Internal Structure*: CFA was performed on the DEBQ items, and the results indicated a three‐factor structure. Model fit was assessed using the chi‐square to degrees of freedom ratio and the RMSEA. *Evidence Based on Relations with Other Variables (Convergent and Discriminant)*: Factorial Invariance for age was tested, along with multi‐group tests for age, sex and BMI status. Mean and standard deviation scores were used in the scales and compared according to sex, BMI and age (65–73 years).
4	Specific Age‐ and Gender‐Specific Norms for the German Version of the Three‐Factor Eating Questionnaire (TFEQ) Löffler et al. (2015) Appetite	Provide updated age‐ and gender‐specific normative data for the German version of the TFEQ—‘Fragebogen zum Essverhalten’ (FEV)—focusing particularly on a representative sample of middle‐aged and older individuals (40–79 years). Germany 3144 individuals (1639 men) aged 40–79 years (54.97 ± 9.70).	FEV is a multidimensional instrument that assesses eating behaviour through 51 items measuring three dimensions: restrained eating (21 items), disinhibition (16 items) and hunger (14 items). Response scale: Dichotomous, where each item is scored as 0 or 1 point. The maximum score for the cognitive restraint domain is 21 points, for disinhibition is 16 points, and for hunger is 14 points. Higher scores indicate stronger characteristic values in the respective domain. Self‐administered: The administrator presents the printed instrument but does not assist in its completion. Tripartite Model.	*Evidence Based on Content*: The results from this stage of the cross‐cultural adaptation study to German were used; however, the data are not presented in the studies. *Evidence Based on Internal Structure*: Based on the study Westenhoefer et al. [48]. Cronbach's *α* ranged from 0.7 to 0.8. The type of factor analysis used was not mentioned. A correlation was found between the disinhibition domain and the hunger domain. *Evidence Based on Relations with Other Variables (Convergent and Discriminant)*: Group differences in eating behaviour problems were analysed using the chi‐square test. A Univariate Analysis of Variance assessed the effect of age and gender across the three FEV domains, with partial *η* ^2^ values calculated for effect sizes. Gender had significant effects in all three domains, with higher mean scores in women. For cognitive restraint, disinhibition and hunger domains, the *F* values were 155.5 (*p* < 0.001), 68.2 (*p* < 0.001) and 18.7 (*p* < 0.001), respectively, with partial *η* ^2^ values of 0.05, 0.02 and 0.01, indicating moderate to substantial effects. For age, significant effects were also observed. In cognitive restraint, both sexes scored higher in older age groups. In disinhibition and hunger, older participants scored significantly lower. *F* values were 53.0 (*p* < 0.001), 15.2 (*p* < 0.001) and 6.6 (*p* < 0.001), with partial *η* ^2^ values of 0.05, 0.014 and 0.01, indicating moderate effects. Women showed a significantly higher frequency of eating‐related problems, especially cravings for sweets. Younger participants, regardless of sex, reported eating in response to stress more frequently than older participants.
5	Does Food Addiction Contribute to Excess Weight Among Clinic Patients Seeking Weight Reduction? Examination of the Modified Yale Food Addiction Scale. Masheb et al. (2018) Comprehensive Psychiatry	Determine the prevalence rate of diagnosed food addiction, as measured by the Modified Yale Food Addiction Survey—Short Version (mYFAS), in a sample of veterans seeking treatment for weight management. Examine the convergent validity of the mYFAS in this sample by analysing correlations between the mYFAS and measures of weight, eating behaviour, mental health and other behaviours, as well as the co‐occurrence of food addiction with Binge Eating Disorder (BED). Additionally, assess whether food addiction and body mass index (BMI) are related, and test the incremental validity of food addiction in explaining unique variance in BMI beyond relevant psychosocial correlates. USA 126 individuals (113 men) with a mean age of 61.8 ± 8.6.	mYFAS is a unidimensional instrument that assesses food consumption with characteristics similar to addiction. It uses survey items aligned with the diagnostic criteria for substance dependence as outlined in the Diagnostic and Statistical Manual of Mental Disorders–IV–Text Revision (DSM‐IV‐TR). The instrument consists of nine items. Response scale: Likert‐type scale from 1 to 7 points (1 = ‘never’ to 7 = ‘every day’) for Questions 1–7 and a dichotomous response scale (0 or 1) for Questions 8 and 9. To meet the diagnostic threshold criteria, participants must reach the cutoff values for at least three of the questions (1–5 and 8–9), and for question 6 or 7. Self‐report: The administrator presents the printed instrument, and the volunteer completes it without assistance. Tripartite Model.	*Evidence Based on Content*: Not provided. *Evidence Based on Internal Structure*: Demographic information (e.g., age, gender and race/ethnicity) and food addiction screening were analysed using basic means, standard deviations and frequency counts. Chi‐square tests were used to evaluate the diagnostic overlap between food addiction and Binge Eating Disorder (BED). The chi‐square test revealed a significant overlap between BED and diagnosed food addiction (*χ* ^2^ (1) = 40.2, *p* < 0.001). *Evidence Based on Relations with Other Variables (Convergent and Discriminant)*: Convergent validity: Correlations between mYFAS scores and measures of weight and eating behaviours (BMI, general eating pathology, emotional eating and night eating), as well as mental health and behavioural measures (screening for depression, insomnia, Posttraumatic Stress Disorder [PTSD] and alcohol use disorders). Step 1: Eating pathology uniquely explained 5.6% of the variance in BMI. Step 2: When food addiction was added, it accounted for an additional 15.1% of the variance in BMI. Overall, both predictors together explained 20.8% of the variance in BMI.
6	The Mindful Eating Behavior Scale: Development and Psychometric Properties in a Sample of Dutch Adults Aged 55 Years or Older. Winkens et al. (2017) Journal of the Academy of Nutrition and Dietetics	Develop a new scale to measure the attentional element of mindful eating, test its internal structure and reliability, and assess whether mindful eating is metrically distinguishable from other eating styles. Netherlands 1277 individuals (661 women) aged 55 and older (66.8 ± 8.1).	Mindful Eating Behavior Scale (MEBS) is a multidimensional instrument, shorter than other versions, designed for use in healthcare settings where time constraints exist for assessing mindful attention to eating behaviour. It consists of 17 items measuring the domains of mindful eating separately from emotional, external and restrained eating. Response scale: 5‐point Likert scale (1 = ‘never’ to 5 = ‘very frequently’). Self‐report: The administrator presents the instrument online or in print, and the volunteer completes it without assistance. Tripartite Model.	*Evidence Based on Content*: The MEBS was developed by selecting 20 items from existing instruments: 2 from the Distraction domain (MEQ—Framson et al. [49]), 5 from Awareness, 3 from Acting with Awareness and 2 from Disorganised Eating (MES—Hulbert‐Williams et al. [50]) and 6 from Hunger & Satiety Cues (IES‐2—Tylka et al. [51]). Two additional items were created: ‘I watch TV while eating’ and ‘I read while eating’. Items were translated into Dutch and back‐translated into English. A pilot study (18 participants) tested comprehensibility. The 20‐item scale used a 1 ‘never’ to 5 ‘very frequently’ response scale. Expected domains: Eating with Focus, Eating While Paying Attention to Hunger and Satiety Cues, Awareness of Eating and Eating Without Distraction. *Evidence Based on Internal Structure*: Structural equation modelling (SEM), reliability and measurement invariance were tested across gender, age and BMI groups. Configural, metric, scalar and strict invariance were examined, with CFI decrease < 0.01 and RMSEA increase < 0.015 as thresholds. The final ESEM model (Model 3) met all fit criteria, and the CFA model (M4) also showed good fit. All item loadings were ≥ 0.65, except Item 20 (0.39). Factor correlations: moderate between Eating with Focus & Eating with Awareness (*r* = 0.39), small‐to‐moderate between Eating with Focus & Hunger & Satiety Cues (*r* = 0.25) and Eating with Focus & Eating Without Distraction (*r* = 0.20), strong between Eating with Awareness & Eating Without Distraction (*r* = 0.51) and small for Hunger & Satiety Cues & Eating Awareness (*r* = 0.03) and Eating Without Distraction (*r* = 0.14). Internal consistency (Cronbach's *α*): Eating with Focus (0.85), Hunger & Satiety Cues (0.89), Eating Awareness (0.81) and eating Without Distraction (0.70). Model fit: CFI = 0.95, TLI = 0.93 and RMSEA = 0.03. Measurement invariance was satisfactory across gender, age and BMI, with minimal changes in fit. Strict invariance for age showed a CFI change of 0.01. Modification indices linked variance issues to Item 1 (I notice flavours and textures when eating my food). Adjusting its error variance improved CFI to 0.96 (ΔCFI = 0.003). *Evidence Based on Relations with Other Variables (Convergent and Discriminant)*: Most correlations were low ( < 0.2) or moderate (0.2–0.3), indicating good preliminary convergent validity. Eating with Focus, Eating with Awareness and Eating Without Distraction correlated positively with self‐esteem, life satisfaction and weight satisfaction, and negatively with depressive symptoms, difficulty identifying and describing feelings, and perceived stress. Hunger & Satiety Cues correlated positively with weight satisfaction and negatively with difficulty describing feelings and BMI. Maximum correlations: Eating with Focus |*r*| = 0.25 (difficulty identifying/describing feelings), Hunger & Satiety Cues |*r*| = 0.12 (External Eating, BMI), Eating Awareness |*r*| = −0.32 (Emotional Eating) and Eating Without Distraction |*r*| = −0.28 (Emotional Eating). Unexpectedly, Eating with Focus did not correlate with Emotional/External Eating but correlated positively with Restrained Eating. Hunger & Satiety Cues showed a positive relationship with External Eating, and Eating Without Distraction did not correlate with BMI.
7	Food Avoidance Beliefs and Behaviors Among Chinese Cancer Patients: Validation of a New Measurement Tool. Yung et al. (2019) Journal of Nutrition Education and Behavior	Develop and validate the Cancer Patient Food Avoidance Beliefs Scale (CPFAB), investigating dietary attitudes and food avoidance practices among Chinese cancer patients, influenced by Traditional Chinese Medicine (TCM), to understand the implications for nutrition and well‐being in this population. Hong Kong 245 individuals (142 men) aged 49–69 years.	CPFAB is a multidimensional instrument designed to measure food avoidance beliefs among cancer patients. It is divided into three dimensions of Food Avoidance Behaviors (FABs): (1) Avoiding Specific Food Items (CPFAB‐SF)—refraining from consuming foods perceived as harmful; (2) Avoiding Nutritious Foods (CPFAB‐NF)—reducing intake of foods perceived as highly nutritious; and (3) Overall Food Intake Reduction (CPFAB‐OA)—decreasing total intake to deliberately deprive the body of food. Response scale: 5‐point Likert scale, ranging from 1 ‘strongly disagree’ to 5 ‘strongly agree’. A higher score reflects a stronger belief that FABs are beneficial for cancer patients (total summative score for 15 items = 1575 points). Hetero‐report: The administrator presents the printed instrument and assists with its completion. Tripartite Model.	*Evidence Based on Content*: The CPFAB was developed based on discussions in three focus groups with distinct characteristics. Based on the findings from these groups, the research team designed a questionnaire. The scale items corresponded to three dimensions of FABs, with a total of 15 items: (1) Avoiding Specific Food Items (refraining from consuming foods perceived as harmful by the participant); (2) Avoiding Nutritious Foods (reducing foods perceived as highly nutritious); and (3) Overall Food Intake Reduction (decreasing food intake with the intention of depriving the body). *Evidence Based on Internal Structure*: Items were analysed for reliability and test–retest consistency. *Evidence Based on Relations with Other Variables (Convergent and Discriminant)*: Logistic regression analyses were conducted to establish concurrent validity. The CPFAB‐SF subscale showed a significant association with participants' intention to reduce the consumption of specific food items and nutritious foods (OR = 1.15; 95% CI = 1.04–1.28), as well as with actual avoidance of more than four food items. The CPFAB‐NF subscale was significantly associated with almost all variables, except for the intention to reduce the consumption of specific food items and the recommended energy intake level. The CPFAB‐OA subscale was associated with participants' intention to consume less nutritious foods and a reduction in overall food intake.
8	Development and Psychometric Testing of the Mealtime Engagement Scale for Direct Care Staff in Nursing Homes for Residents with Dementia Liu et al. (2021)	To develop the Mealtime Engagement Scale (MES) and test its reliability (internal consistency, inter‐rater reliability and intra‐rater reliability) and validity (content validity, concurrent validity and convergent validity). USA Seven individuals (six men) over 60 years old (88.12 ± 9.45).	MES is a unidimensional instrument composed of 18 items that assess various aspects of caregiver engagement during mealtimes. These assessments include identifying differences in mealtime engagement based on the characteristics of both caregivers and residents. Response scale: Likert‐type scale from 0 to 6 (0 = ‘never’ [0 times], 1 = ‘sometimes’ [1–2 times], 2 = ‘frequently’ [3–5 times], 3 = ‘always’ [6 times or more]). Hetero‐report in paper format: The administrator watches a video of the resident eating with caregiver assistance and completes the questionnaire accordingly. Tripartite Model.	*Evidence Based on Content*: The MES was developed through a literature review to establish theoretical foundations and study records, expert reviews to generate and refine item sets, establish face and content validity, and a pre‐test to develop response options and improve scale feasibility using video observations of mealtime interactions from a dementia communication trial. For the 26‐item set, the item‐level content validity index (I‐CVI) ranged from 0.67 to 1.00 (feasibility) and from 0.83 to 1.00 (relevance, specificity and clarity/readability). The Scale‐Level Content Validity Index/Average (S‐CVI/Ave) was 0.98 (relevance), 0.96 (specificity), 0.98 (clarity/readability) and 0.96 (feasibility). The S‐CVI was 1.00 for all criteria based on the frequency range (0–7). *Evidence Based on Internal Structure*: Item analysis, reliability and inter‐rater reliability were conducted. *Evidence Based on Relations with Other Variables (Convergent and Discriminant)*: Concurrent validity was assessed using Spearman's correlation between the total MES score and the total score on the Relational Behavior Scale (RBS). Convergent validity was examined through Spearman's correlation between the total MES score and the total score on the Relational Care Checklist during Mealtimes for individual residents.

Abbreviations: CI, confidence interval; ESEM, exploratory structural equation modelling approach; OR, odds ratio; TLI, Tucker–Lewis Index; USA, United States of America.

**Table 2:** Percentage mapping of the studies included in the review.

Characteristics	*N* (%)
Instrument name	
Three‐Factor Eating Questionnaire‐R18 (TFEQ‐R18)	1 (12.5)
Modified Yale Food Addiction Scale Version 2.0 (MYFAS‐2.0)	1 (12.5)
Modified Yale Food Addiction Scale (MYFAS)	1 (12.5)
Three‐Factor Eating Questionnaire‐R51 (TFEQ‐51)	1 (12.5)
The Dutch Eating Behaviour Questionnaire (DEBQ)	1 (12.5)
Mindful Eating Behavior Scale (MEBS)	1 (12.5)
Food Avoidance Beliefs and Behaviors (FABB)	1 (12.5)
Mealtime Engagement Scale (MES)	1 (12.5)
Continent of origin	
Europe	5 (71.43)
North America	1 (14.29)
Asia	1 (14.29)
Objective	
Development	4 (50)
Adaptation	4 (50)
Development model	
Tripartite	8 (100)
Standards	0 (0)
Response model	
Ordinal	7 (87.5)
Dichotomous	1 (12.5)
Content‐based evidence	
Present	4 (50)
Not present	4 (50)
Internal structure‐based evidence	
Present	8 (100)
Not present	0 (0)
Evidence based on relationships with other variables	
Present	8 (100)
Not present	0 (0)

Though no instrument was mentioned more than once in Table 2, the Three‐Factor Eating Questionnaire (TFEQ) and the Yale Food Addiction Scale (YFAS) appeared in different versions. The TFEQ, assessing general EB, was used in two adaptations: Löffler et al. [26] employed the original version (51 items, focusing on cognitive restraint, disinhibition and hunger); Malkki‐Keinänen et al. [25] used a more recent version (18 items, assessing cognitive restraint, emotional eating and uncontrolled eating) (Table 1).

Similarly, the YFAS, assessing food addiction, also had two versions: Masheb et al. [28] used the version based on DSM‐IV‐TR criteria; Pipová et al. [29] applied a more recent version adapted to updated DSM‐5 criteria (Table 1). Other identified instruments—The DEBQ, Mindful Eating Behavior Scale (MEBS), Food Avoidance Beliefs and Behaviors (FABB) and Mealtime Engagement Scale (MES)—assessed, respectively, eating motivations, mindful eating, avoidance behaviours in cancer patients and caregiver engagement during meals.

Europe stood out as the continent with the highest percentage of instruments adapted or developed for the population, at 62% (5) (Table 2). Article objectives regarding instrument development or adaptation were equally distributed: 4 articles (50%) focused on development and 4 (50%) on adaptation (Table 1). The most used response scale was the ordinal Likert type, present in 87% (7) of articles. The Tripartite model was the sole framework used for instrument adaptation or development, representing 100% (8) of articles (Table 2). Content‐based evidence was presented in only half of the articles (4, 50%), while all articles demonstrated evidence based on internal structure and relationships with other variables (Table 2).

In COSMIN analysis (Table 3), all articles show a considerable proportion of inadequate items, especially regarding instrument design (Box 1), structural validity (Box 3), internal consistency (Box 4) and measurement errors (Box 7). For content validity (Box 2), TFEQ‐R18, MYFAS‐2.0 and MYFAS instruments were inadequate. DEBQ, TFEQ‐R21, MEBS, FABB and MES instruments were doubtful. For cross‐cultural validity (Box 5), reliability (Box 6), criterion validity (Box 8) and hypothesis testing content validity (Box 9), articles ranged from inadequate to very good, as per Table 3. A detailed explanation of the authors' reasons for COSMIN classifications is in Supporting Information S1.

**Table 3:** Assessment of bias risk and psychometric qualities.

Instruments/author	Box 1	Box 2	Box 3	Box 4	Box 5	Box 6	Box 7	Box 8	Box 9
1. Three‐Factor Eating Questionnaire‐R18 (TFEQ‐18)									
Malkki‐Keinänen et al. [25]	I	I	I	I	D	I	I	VG	A
2. Modified Yale Food Addiction Scale Version 2.0 (MYFAS‐2.0)									
Pipová et al. [29]	I	I	I	I	D	I	I	I	VG
3. Dutch Eating Behaviour Questionnaire (DEBQ)									
Bailly et al. [27]	I	D	I	I	I	I	I	I	VG
4. Three‐Factor Eating Questionnaire‐R151 (TFEQ‐51)									
Löffler et al. [26]	I	D	I	I	D	I	I	I	VG
5. Modified Yale Food Addiction Scale (MYFAS)									
Masheb et al. [28]	I	I	I	I	D	I	I	VG	VG
6. Mindful Eating Behavior Scale (MEBS)									
Winkens et al. [33]	I	D	I	I	D	I	I	VG	VG
7. Food Avoidance Beliefs and Behaviors (FABB)									
Yung et al. [30]	I	D	I	I	D	VG	I	I	VG
8. Mealtime Engagement Scale (MES)									
Liu et al. [31]	I	D	I	I	I	A	I	VG	D

Abbreviations: A, adequate; Box 1, instrument development; Box 2, content validity; Box 3, internal validity; Box 4, internal consistency; Box 5, cross‐cultural validity/measurement invariance; Box 6, reliability; Box 7, measurement errors; Box 8, criterion validity; Box 9, hypothesis testing for construct validity; D, doubtful; I, inadequate; NA, not applicable; VG, very good.

## 4 Discussion

This review identified five valid instruments for measuring EB in late adulthood, predominantly from Europe (71.43%). All were adapted or developed using the Tripartite model, with psychometric properties ranging from questionable to good.

The geographical concentration of EB assessment instruments, with psychometric qualities primarily tested in Europe, aligns with UN (2024) projections of Europe having the highest growth in individuals over 60 years of age [1]. This concentration may be attributed to many European nations having already reached population peaks, increasing demand for tailored studies and instruments [1]. Conversely, the low representation from Latin America and Asia (14.29%) highlights a significant gap, especially given these regions also face ageing challenges, as noted in the UN report (2024). Generalising psychometric findings from a single region is not advisable, as cultural and demographic variability can significantly influence results [23]. Cross‐cultural adaptation and validation of measurement instruments are essential to ensure cultural differences are adequately captured and psychometric outcomes are valid and unbiased across different contexts [24]. Therefore, a lack of global representation in psychometric instruments may compromise applicability in diverse ageing populations like Latin America and Asia [23].

Analysis of the eight instruments revealed both overlaps and divergences in assessing EB in older people. The TFEQ‐R18 [25], TFEQ‐51 [26] and DEBQ [27] largely converge on conscious control, emotional response and environmental influences on food intake. However, the TFEQ‐5131 assesses cognitive restraint, hunger and disinhibition concepts, which were updated by Karlsson et al. [11] in the TFEQ‐18 to assess cognitive restraint, emotional eating, and uncontrolled eating. Löffler et al. [26] used the older TFEQ‐51, which Karlsson et al. [11] considered inconsistent in its three‐factor factorial structure when applied to large samples of obese individuals. This instability was also observed in normal‐weight samples, where disinhibition and hunger factors showed inconsistent item groupings. The TFEQ‐18 version proved consistent across genders, different age groups and BMI classes within the obese sample, offering a more precise and structurally valid measure for key EB dimensions. Therefore, for adaptation, the TFEQ‐18 is considered more robust and appropriate, especially in contexts where the obese population is the focus, due to its proven validity and replicability.

The DEBQ also assesses emotional eating and cognitive restraint, and uniquely, external eating. However, its proprietary nature makes adaptation for other contexts and scientific research difficult. In contrast, the MYFAS [28] and MYFAS‐2.0 [29] address specific addiction topics, formulating questions about loss of control (When I start eating certain foods, I end up eating much more than I planned), withdrawal symptoms (I get agitated when I stop eating certain foods) and functional impairment (My eating habits cause significant distress). The MEBS introduces unique topics related to awareness during eating, with questions about mindfulness (I notice flavours and textures when I eat), recognition of bodily cues (I trust my body to tell me when to stop eating) and mental presence during meals (‘I eat without being aware of what I am doing’—reverse scoring).

Completely diverging, the FABB [30] addresses specific food avoidance topics in oncological contexts, questioning beliefs about harmful foods (Certain foods can worsen my condition), nutritional restrictions (I should avoid nutritious foods during treatment) and intentional intake reduction (It's better to eat less so as not to feed the cancer). The MES [31] represents a unique caregiver‐centred approach, addressing social interaction during meals (The caregiver offers food choices), non‐verbal communication (The caregiver maintains eye contact during eating) and emotional support (The caregiver shows patience when the resident eats slowly). Although some instruments ask conceptually similar questions about emotional and cognitive aspects of eating, most address distinct and specific topics for different clinical contexts, highlighting the need for greater clarity on which aspects of EB are priorities for assessment in populations aged 60 or more.

The selection of the appropriate psychometric instrument is fundamental to ensure the target construct is accurately assessed, especially in clinical and population contexts [7]. When construct validity is severely neglected due to absent essential measurement information, methodological differences between assessments and problems in translation and cultural adaptation, the reliability and validity of the evidence found are compromised, weakening the knowledge base about the construct and limiting the instrument's ability to measure interventions and/or diagnose pathologies, given that its results do not actually reflect what is intended to be measured [32].

Regarding the development model, all eight articles applied the Tripartite model, representing 100% of the analysed studies. Since 2000, Teresi, Kleinman and Ocepek‐Welikson have discussed a new model for instrument adaptation and development. Since the release of the Standards (2014), this model has been recommended for psychometric measurement development and adaptation due to its robust, precise, and less biased approach, ensuring invariant sections independent of the sample [7, 33]. This model primarily utilises Item Response Theory (IRT), a mathematical model in psychometrics that evaluates how individuals' responses in a test relate to specific item characteristics, such as difficulty, discrimination and guessing [30].

Unlike the Tripartite model, IRT allows detailed analysis of each item, enabling more precise and individualised assessments of respondents' psychological traits or abilities [30]. Additionally, IRT facilitates adaptive testing, where items are tailored to each person's ability level, yielding more consistent and comparable results, even when different sets of items are applied. This enhances the accuracy and fairness of psychological assessments, reduces bias and optimises test validity [30].

A central issue in psychometric studies is the choice between developing or adapting instruments. This choice must be clearly justified based on specific research needs and cultural context, mitigating validity loss risks and ensuring greater relevance to the target population [7]. The lack of justification in the analysed studies may reflect resource limitations or an underestimation of rigorous adaptation or development importance [34]. The findings in this review indicate an equal distribution between development and adaptation approaches (50% for each); however, none of the articles justified their chosen approach. This omission, along with inadequate refinement procedures like pilot testing, results in methodologically weak instruments that may not effectively represent target constructs, making EB evaluation uncertain [7, 35].

In the COSMIN analysis (Table 3), all instruments were classified as ‘inadequate’ in Box 1 (instrument development) due to absent or failed pilot studies (not mentioned or reported, or unrepresentative sample) and inappropriate sample sizes for statistical analyses (Supporting Information S1). The study by Terwee et al. [35], which established the COSMIN standards through an international Delphi consensus, highlights pilot testing and cognitive interviews as indispensable steps to ensure item comprehensibility and relevance for the target population. Neglecting this phase, as observed in the analysed studies, invalidates the premise that the instrument effectively measures the desired construct. Consequently, the construct validity of these instruments is questionable, compromising all other psychometric properties, including structural validity and internal consistency [36]. The practical implications are serious: instruments with fragile factorial structure can lead to low discriminative power, incorrect diagnoses and imprecise assessments of intervention effectiveness [32]. This cascade of failures, originating in the development phase, compromises not only data quality but also the external reliability and clinical utility of the evidence generated [32, 36].

The selection of response scales in a questionnaire is crucial to ensuring the precision and validity of collected data, as it directly influences how respondents perceive and express their answers [37, 38]. In the identified instruments, 87% of the articles used an ordinal Likert‐type scale. This prevalence aligns with the literature, as this model is widely used in behavioural contexts due to its simplicity and ability to quantify nuances of opinion. [37, 38]. The design of instruments influences item clarity and cognitive load reduction, essential for data quality [39]. For participants over 60 years old, who may experience greater cognitive overload or less familiarity with assessment instruments, scales with many response options can be confusing [39]. Thus, the predominant choice for the Likert scale in the analysed studies, more than a mere convention, represents a methodologically robust approach aligned with good practices for assessing vulnerable populations.

During the qualitative analysis of content‐based evidence, only 50% of the included studies presented this step (Table 2), despite content validity being a fundamental phase in instrument development that adequately reflects the constructs they aim to measure [40]. This process involves consulting experts who evaluate item representativeness, clarity and comprehensiveness, as well as conducting pilot studies to refine the instrument before applying it to a larger sample [7, 40].

This qualitative observation on content analysis aligns with the COSMIN classifications, in which many evaluated studies did not follow appropriate procedures for this phase. The instruments TFEQ‐18 [25], MYFAS‐2.0 [29] and MYFAS [28] were rated as ‘inadequate’ in Box 2 (Content Validity). This rating stemmed from a lack of transparency in target population data analysis, reports of only one interviewer being involved in data collection, or an insufficient sample size to support robust statistical analysis (Supporting Information S1).

These methodological limitations are directly addressed by the COSMIN standards, which stress the importance of a detailed, standardised and transparent approach to content validity assessment [36]. The absence of multiple reviewers during qualitative data collection and analysis undermines objectivity and reliability, while an inadequate sample size precludes statistically robust analyses capable of confirming item appropriateness [36]. As a result, the content validity of these instruments is compromised, raising concerns about their ability to accurately capture the nuances of EB among adults aged 60 and over—including critical factors such as age‐related changes in appetite, medication use and sensory alterations [36].

The COSMIN framework highlights that content validity encompasses not only item relevance but also their comprehensibility and comprehensiveness for the intended population, criteria that, in this instance, appear not to have been adequately addressed [36]. Other instruments were rated as having ‘doubtful’ content validity due to insufficient information on how this stage of development was conducted, the type of analysis applied, the number of interviewers involved and an inadequate sample size to support the analysis [36]. This ‘doubtful’ rating reflects the lack of sufficient detail required for a rigorous evaluation, which, according to COSMIN, prevents a conclusive judgement of evidence quality and therefore limits recommendations for the instrument's clinical or research use.

In the qualitative analysis, 100% of the instruments demonstrated internal structural validity (Table 2). However, COSMIN results showed that all instruments were rated as ‘inadequate’ in Box 3 (Internal Validity) and Box 4 (Internal Consistency) (Table 3). While Cronbach's alpha was used to measure internal consistency and exploratory and/or confirmatory factor analyses to identify and confirm underlying factor structures, robust practices such as IRT were not applied. IRT offers significant advantages for addressing measurement errors but requires larger samples, which many studies fail to achieve. Additionally, while IRT is integral to the Standards development model, all identified instruments were developed or adapted using the Tripartite model, as previously noted, potentially explaining the lack of IRT application [7, 41, 42]. Consequently, while traditional structural analysis techniques are consistently used, the absence of robust methodologies like IRT suggests potential inadequacy in the rigour and precision of the results.

The validity of an instrument is not an abstract and universal property; it depends on the interaction between the items and the characteristics of the target population [43]. As argued in rigorous methodological contexts, specific factors of a group or context can pose serious threats to the validity of conclusions if not properly controlled [43]. In the case of the elderly, cognitive and behavioural characteristics related to EB may differ substantially from those of other age groups. Traditional factor analysis may not be sensitive enough to identify if an item functions differently in this group (known as differential item functioning, or DIF), something that IRT is designed to identify [43]. Without this deeper analysis, there is a risk of using instruments whose internal structure, although seemingly valid for the general population, is inadequate and potentially biased for the elderly, leading to erroneous clinical interpretations and conclusions [43].

The validity of cross‐cultural adaptation or measurement invariance (Box 5) showed that the DEBQ [27] and MES [31] were inadequate (Table 2 and Supporting Information S1). The other instruments were classified as doubtful because, despite achieving a good sample size for analysis, MGCFA, regression analysis or DIF was not performed (Table 2 and Supporting Information S1). The other instruments were classified as doubtful because, despite achieving a good sample size for analysis, MGCFA, regression analysis or DIF was not performed (Table 2 and Supporting Information S1). The absence of such analyses compromises validity. The other instruments were classified as doubtful, because despite achieving a good sample size for analysis, MGCFA, regression analysis or DIF was not performed (Table 2 and Supporting Information S1). The absence of analyses such as MGCFA and, more specifically, techniques to detect DIF, compromises the validity of any comparison between the instrument's original population and the elderly population. DIF occurs when individuals from different groups, with the same level of the latent trait, respond systematically differently to an item, which represents a serious ‘threat to test fairness’ [44].

Without verifying the absence of DIF, we cannot guarantee that differences in scores between young and old are due to real differences in EB and not to a bias of the instrument itself. Regression analysis, described as a ‘powerful and versatile instrument’ in research [45], is one of the standard tools for investigating DIF [44]. Its non‐application in the studies classified as ‘doubtful’ means that the authors did not verify whether belonging to the age group (being elderly) influenced the probability of responding to the items, even controlling for the general level of EB. This omission prevents the confirmation that the instrument functions equivalently between groups, making any direct comparison of scores scientifically unsustainable and potentially misleading [44, 45].

In Box 6, instrument reliability is analysed. Most articles were classified as ‘inadequate’ for failing to clarify the use of the intraclass correlation coefficient (ICC), not specifying the time interval between tests or not using Pearson and Spearman correlation. The FABB [30] was classified as very good, and the MES as adequate. This is because during the construction of the FABB, there were no methodological failures. In the MES, despite assuming that the participants were stable and under the same conditions in the test–retest, they did not explain how this was determined (Table 2 and Supporting Information S1).

The ICC measures the consistency of responses among raters or across different occasions, indicating the degree to which observed variations reflect true differences between individuals rather than measurement errors. The absence of ICC makes instrument reliability questionable, as ICC distinguishes real participant variations from those attributable to random errors or inconsistencies in the instrument, suggesting a lack of precision and limiting its applicability in contexts requiring high reliability for clinical or research use [7].

Regarding the assessment of measurement error (Box 7), all instruments were rated as ‘inadequate’ due to non‐equivalent test conditions, the absence of standard error of measurement (SEM) calculations, and reliance solely on Cronbach's alpha. While Cronbach's alpha is a measure of internal consistency, it does not directly inform the precision of an individual's score. SEM, on the other hand, quantifies ‘the spread of measurement errors when estimating an examinee's true score from their observed score.’ [46] Using SEM allows for the construction of a confidence interval around an individual's observed score, providing an estimate of the margin of error associated with that measurement [46].

By reporting only Cronbach's alpha, the authors of the studies analysed in this review omitted a critical next step: translating test reliability into a clinically interpretable error metric [46]. Without SEM, it is impossible to determine how precise an individual's score is. For example, a score of 15 on an EB instrument could reflect a true score ranging anywhere from 10 to 20, depending on the SEM [46]. This level of uncertainty is particularly important in clinical decision‐making, especially for older adults, where individual variability tends to be high [46].

Evidence based on relationships with other variables was reported in 100% of the analysed articles (Table 2). However, in the COSMIN assessment (Box 8), instruments such as the MYFAS‐2.0 [29], DEBQ [27], TFEQ‐51 [26] and FABB [30] were rated as ‘inadequate’ for failing to establish correlations with other variables. The mere presence of statistical relationships in validation studies does not guarantee that such relationships were established in line with contemporary construct validity standards [7]. These standards require theoretical justification and alignment with the nomological network of the construct [7].

Evidence based on relations to other variables must demonstrate not only statistically significant correlations but also theoretical and predictive coherence with other instruments, behaviours, or known groups [36]. Therefore, studies that fail to conduct appropriate convergent, discriminant, or predictive analyses may compromise construct validity, even if other forms of psychometric evidence are present [36].

Hypothesis testing for construct validity (Box 9) received the most positive evaluations in the COSMIN analysis. Almost all instruments were rated as ‘very good,’ indicating that the original studies employed high‐quality methodologies in assessing their psychometric properties. This rating suggests that authors formulated clear, justified a priori hypotheses, selected appropriate comparator instruments and applied correct statistical analyses in accordance with best practices in construct validation [36].

However, two notable exceptions were the TFEQ‐18 and the MES. The TFEQ‐18 was rated as ‘adequate’ rather than ‘very good.’ The shortcoming here was not in execution but in reporting: while subgroup characteristics were well described, the authors did not provide sufficient detail regarding the statistical analyses performed, instead assuming their appropriateness [36]. According to COSMIN methodology, complete transparency is required for the highest rating [36].

The MES received an even lower rating of ‘doubtful.’ This was due to poor or missing descriptions of important characteristics of the subgroups in which the instrument was tested [36]. Construct validity assessment is highly context‐dependent. Without a clear description of the sample, it is impossible for reviewers to determine whether the hypotheses and comparator instruments were relevant and appropriate—thus compromising confidence in the results and justifying the ‘doubtful’ rating [36].

The limitations of this systematic review include the restriction of the search to a limited number of databases (Scopus, ScienceDirect, PsycINFO and Medline) and to articles published exclusively in English, Spanish or Portuguese. Although these databases are comprehensive, the exclusion of other sources and languages may have led to the omission of relevant studies, introducing potential selection bias. Additionally, the temporal delimitation of the search from the year 2000 onwards—while justified by the growing interest in the construct—may have excluded earlier research that could provide a more comprehensive perspective. Finally, the composition of the review team, with varying levels of experience, and the assumption that the absence of information in primary articles equates to non‐performance of certain steps, may have introduced information and data extraction bias, despite efforts to ensure impartiality and consensus.

Although eight instruments exist for assessing EBs in individuals aged 60 and over, the methodological robustness of all of them is questionable, according to the criteria used for this review. A detailed analysis of their psychometric properties reveals consistent shortcomings in critical areas such as instrument development (Box 1), content validity (Box 2), internal validity (Box 3) and internal consistency (Box 4). Even those instruments that demonstrated some ‘Adequate’ or ‘Very Good’ ratings in other domains (e.g., criterion validity, hypothesis testing for construct validity) exhibit fundamental weaknesses that compromise their overall precision and reliability for the target population. The predominance of European‐based studies also limits the cross‐cultural applicability of these instruments, highlighting the urgent need for more rigorous and culturally sensitive approaches to the development and adaptation of tools for assessing EB in older adults.

## Author Contributions

All authors met the criteria for authorship as defined by the *International Committee of Medical Journal Editors (ICMJE)*, which include: (1) substantial contributions to the conception or design of the work, or the acquisition, analysis, or interpretation of data; (2) drafting the work or revising it critically for important intellectual content; (3) final approval of the version to be published; and (4) agreement to be accountable for all aspects of the work, ensuring the accuracy and integrity of any part of the content. The specific contributions were as follows: Giulia de Pádua Constâncio participated in all stages of the study. Gisele Teixeira de Souza Silva contributed to data collection and assisted in drafting the introduction and methodology sections. Maria Fernanda Ferrari Bahia contributed to data interpretation and assisted in drafting the results and discussion sections. Marcela Rodrigues de Castro contributed to data interpretation, supervised and revised the writing of the entire manuscript. All authors declare that they have reviewed and approved the final version of the manuscript and agree to be responsible for all aspects of the work, ensuring its accuracy and integrity. We affirm that the content of this manuscript is original and has not been published, nor is it under consideration for publication, in any other journal, either in whole or in part.

## Ethics Statement

The study was approved by the Research Ethics Committee of the Federal University of Bahia (Federal University of Bahia, Rua Padre Feijó, no. 312, Casas 47 and 49, Canela District, ZIP Code 40110‐170, Salvador, Bahia, Brazil).

## Conflicts of Interest

The authors declare no conflicts of interest.

## 1 Peer Review

The peer review history for this article is available at https://www.webofscience.com/api/gateway/wos/peer-review/10.1111/jhn.70127.

## Supporting information


**SUPPLEMENTARY MATERIAL 1:** Reasons discussed during the consensus meeting among researchers for the COSMIN classification presented in table 3.

## Data Availability

The data that support the findings of this study are available from the corresponding author upon reasonable request.

## References

[1] United Nations Department of Economic and Social Affairs PD , “World Population Prospects 2024: Summary of Results [Internet],” 2024. www.unpopulation.org.

[2] R. B. Pepe , A. M. Lottenberg , C. T. H. Fujiwara , et al., “Position Statement on Nutrition Therapy for Overweight and Obesity: Nutrition Department of the Brazilian Association for the Study of Obesity and Metabolic Syndrome (ABESO‐2022),” in Diabetology and Metabolic Syndrome, vol. 15 (BioMed Central Ltd, 2023). 10.1186/s13098-023-01037-6 PMC10251611 37296485

[3] M. S. Alvarenga , M. Figueiredo , F. Timerman , and C. M. Antonaccio , Nutrição Comportamental [Behavioral Nutrition], 2nd ed. (Editora Manole Saúde, 2019), 1–624.

[4] M. Alvarengaed , Nutrição comportamental: ciência, prática clínica e comunicação [Behavioral Nutrition: Science, Clinical Practice, and Communication], 1st ed. (Editora Manole Saúde, 2022), 1–224.

[5] A. J. Bukman , A. Ronteltap , and M. Lebrun , “Interpersonal Determinants of Eating Behaviours in Dutch Older Adults Living Independently: A Qualitative Study,” BMC Nutrition 6, no. 1 (December 2020): 55. 33292680 10.1186/s40795-020-00383-2 PMC7656669

[6] G. Caso and R. Vecchio , “Factors Influencing Independent Older Adults (Un)healthy Food Choices: A Systematic Review and Research Agenda,” in Food Research International, vol 158 (Elsevier Ltd, 2022). 10.1016/j.foodres.2022.111476 35840197

[7] AERA, APA, NCME , Standards for Educational and Psychological Testing (American Educational Research Association, 2014), 230.

[8] C. P. Herman and D. Mack , “Restrained and Unrestrained Eating,” Journal of Personality 43 (December 1975): 647–660. 1206453 10.1111/j.1467-6494.1975.tb00727.x

[9] D. B. Allison , L. B. Kalinsky , and B. S. Gorman , “A Comparison of the Psychometric Properties of Three Measures of Dietary Restraint,” Psychological Assessment 4, no. 3 (March 1992): 391–398.

[10] A. J. Stunkard and S. Messick , “The Three‐Factor Eating Questionnaire to Measure Dietary Restraint, Disinhibition and Hunger,” Journal of Psychosomatic Research 29, no. 1 (August 1985): 71–83. 3981480 10.1016/0022-3999(85)90010-8

[11] J. Karlsson , L. O. Persson , L. Sjöström , and M. Sullivan , “Psychometric Properties and Factor Structure of the Three‐Factor Eating Questionnaire (TFEQ) in Obese Men and Women. Results From the Swedish Obese Subjects (SOS) Study,” International Journal of Obesity 24 (2000): 1715–1725, https://www.nature.com/ijo. 11126230 10.1038/sj.ijo.0801442

[12] A. Brytek‐Matera , R. Rogoza , and K. Czepczor‐Bernat , “The Three‐Factor Eating Questionnaire‐R18 Polish Version: Factor Structure Analysis Among Normal Weight and Obese Adult Women,” Archives of Psychiatry and Psychotherapy 19, no. 3 (September 2017): 81–90.

[13] J. C. Cappelleri , A. G. Bushmakin , R. A. Gerber , et al., “Psychometric Analysis of the Three‐Factor Eating Questionnaire‐R21: Results From a Large Diverse Sample of Obese and Non‐Obese Participants,” International Journal of Obesity 33, no. 6 (June 2009): 611–620. 19399021 10.1038/ijo.2009.74

[14] B. G. Martins , W. R. da Silva , J. Maroco , and J. A. D. B. Campos , “Psychometric Characteristics of the Three‐Factor Eating Questionnaire‐18 and Eating Behavior in Undergraduate Students,” Eating and Weight Disorders – Studies on Anorexia, Bulimia and Obesity 26, no. 2 (March 2021): 525–536. 10.1007/s40519-020-00885-9 32166658

[15] E. Kavazidou , M. Proios , I. Liolios , et al., “Structure Validity of the Three‐Factor Eating Questionnaire‐R18 in Greek Population,” Journal of Human Sport and Exercise 7, no. 1 (2012): 218–226.

[16] T. Van Strien , J. E. R. Frijters , G. P. A. Bergers , and P. B. Defares , “The Dutch Eating Behavior Questionnaire (DEBQ) for Assessment of Restrained, Emotional, and External Eating Behavior,” International Journal of Eating Disorders 5, no. 2 (February 1986): 295–315.

[17] V. Viana and S. Sinde , “Estilo alimentar: adaptação e validação do Questionário Holandês do Comportamento Alimentar [Eating Style: Adaptation and Validation of the Dutch Eating Behavior Questionnaire],” 8 (2003): 59–71.

[18] R. M. Baños , A. Cebolla , E. Etchemendy , S. Felipe , P. Rasal , and C. Botella , “Validation of the Dutch Eating Behavior Questionnaire for Children (DEBQ‐C) for Use With Spanish Children,” Nutrición Hospitalaria 26, no. 4 (August 2011): 890–898, http://www.ncbi.nlm.nih.gov/pubmed/22470039. 22470039 10.1590/S0212-16112011000400032

[19] K. Halvarsson and P. O. Sjödén , “Psychometric Properties of the Dutch Eating Behaviour Questionnaire (DEBQ) Among 9±10‐Year‐old Swedish Girls,” European Eating Disorders Review 6, no. 2 (October 1998): 115–125.

[20] M. J. Page , J. E. McKenzie , P. M. Bossuyt , et al., “The PRISMA 2020 Statement: An Updated Guideline for Reporting Systematic Reviews,” BMJ 372, no. 71 (March 2021): 1. 10.1136/bmj.n71 PMC8005924 33782057

[21] J. Muniz , “La validación de los tests [The Validation of Tests],” Metodología de las Ciencias del Comportamiento 5, no. 2 (2004): 121–141.

[22] L. Mokkink , C. A. Prinsen , D. L. Patrick , et al., “COSMIN Study Design Checklist for Patient‐Reported Outcome Measurement Instruments,” 2019, 1–32, www.cosmin.nl.

[23] P. Cruchinho , M. D. López‐Franco , M. L. Capelas , et al., “Translation, Cross‐Cultural Adaptation, and Validation of Measurement Instruments: A Practical Guideline for Novice Researchers,” Journal of Multidisciplinary Healthcare 17 (2024): 2701–2728. 38840704 10.2147/JMDH.S419714 PMC11151507

[24] J. K. Flake , I. J. Davidson , O. Wong , and J. Pek , “Construct Validity and the Validity of Replication Studies: A Systematic Review [Internet],” 2022, https://osf.io/369qj. 10.1037/amp0001006 35482669

[25] K. Malkki‐Keinänen , M. Lankinen , L. Karhunen , and U. Schwab , “Psychometric Evaluation of Three‐Factor Eating Questionnaire‐R18 in Aging Finnish Men With Increased Risk for Type 2 Diabetes,” Nutrition and Health 30, no. 2 (July 2024): 279–290. 35816365 10.1177/02601060221112178 PMC11141102

[26] A. Löffler , T. Luck , F. S. Then , et al., “Age‐ and Gender‐Specific Norms for the German Version of the Three‐Factor Eating‐Questionnaire (TFEQ),” Appetite 91 (August 2015): 241–247. 25889877 10.1016/j.appet.2015.04.044

[27] N. Bailly , I. Maitre , M. Amanda , C. Hervé , and D. Alaphilippe , “The Dutch Eating Behaviour Questionnaire (DEBQ). Assessment of Eating Behaviour in an Aging French Population,” Appetite 59, no. 3 (December 2012): 853–858. 22963738 10.1016/j.appet.2012.08.029

[28] R. M. Masheb , C. B. Ruser , K. M. Min , A. J. Bullock , and L. M. Dorflinger , “Does Food Addiction Contribute to Excess Weight Among Clinic Patients Seeking Weight Reduction? Examination of the Modified Yale Food Addiction Survey,” Comprehensive Psychiatry 84 (July 2018): 1–6. 29654930 10.1016/j.comppsych.2018.03.006

[29] H. Pipová , N. Kaščáková , J. Fürstová , and P. Tavel , “Development of the Modified Yale Food Addiction Scale Version 2.0 Summary Version in a Representative Sample of Czech Population,” Journal of Eating Disorders 8, no. 1 (May 2020): 16. 32391149 10.1186/s40337-020-00292-6 PMC7197132

[30] T. K. C. Yung , J. H. Kim , S. F. Leung , et al., “Food Avoidance Beliefs and Behaviors Among Chinese Cancer Patients: Validation of a New Measurement Tool,” Journal of Nutrition Education and Behavior 51, no. 2 (February 2019): 162–172. 30241704 10.1016/j.jneb.2018.07.009

[31] W. Liu , M. Batchelor , and K. Williams , “Development and Psychometric Testing of the Mealtime Engagement Scale in Direct Care Providers of Nursing Home Residents With Dementia,” Gerontologist 61, no. 8 (December 2021): e410–e420. 32726447 10.1093/geront/gnaa097 PMC8599218

[32] G. O. Boateng , T. B. Neilands , E. A. Frongillo , H. R. Melgar‐Quiñonez , and S. L. Young , “Best Practices for Developing and Validating Scales for Health, Social, and Behavioral Research: A Primer,” Frontiers in Public Health 6 (June 2018): 1–18. 29942800 10.3389/fpubh.2018.00149 PMC6004510

[33] L. H. H. Winkens , T. van Strien , J. R. Barrada , I. A. Brouwer , B. W. J. H. Penninx , and M. Visser , “The Mindful Eating Behavior Scale: Development and Psychometric Properties in a Sample of Dutch Adults Aged 55 Years and Older,” Journal of the Academy of Nutrition and Dietetics 118, no. 7 (July 2018): 1277–1290.e4. 29655657 10.1016/j.jand.2018.01.015

[34] J. K. Flake and E. I. Fried , “Measurement Schmeasurement: Questionable Measurement Practices and How to Avoid Them,” Advances in Methods and Practices in Psychological Science 3, no. 4 (December 2020): 1–10.

[35] C. B. Terwee , C. A. C. Prinsen , A. Chiarotto , et al., “COSMIN Methodology for Evaluating the Content Validity of Patient‐Reported Outcome Measures: A Delphi Study,” in Quality of Life Research, vol. 27 (Springer International Publishing, 2018), 1159–1170. 29550964 10.1007/s11136-018-1829-0 PMC5891557

[36] A. Joshi , S. Kale , S. Chandel , and D. Pal , “Likert Scale: Explored and Explained,” British Journal of Applied Science & Technology 7, no. 4 (February 2015): 396–403.

[37] S. E. Harpe , “How to Analyze Likert and Other Rating Scale Data,” Currents in Pharmacy Teaching and Learning 7, no. 6 (November 2015): 836–850.

[38] A. T. Jebb , V. Ng , and L. Tay , “A Review of Key Likert Scale Development Advances: 1995–2019,” Frontiers in Psychology 12 (2021): 637547. 34017283 10.3389/fpsyg.2021.637547 PMC8129175

[39] A. Roebianto , S. I. Savitri , I. Aulia , A. Suciyana , and L. Mubarokah , “Content Validity: Definition and Procedure of Content Validation in Psychological Research,” Testing, Psychometrics, Methodology in Applied Psychology 30, no. 1 (March 2023): 5–18.

[40] P. J. Ferrando and U. Lorenzo‐Seva , “Assessing the Quality and Appropriateness of Factor Solutions and Factor Score Estimates in Exploratory Item Factor Analysis,” Educational and Psychological Measurement 78, no. 5 (October 2018): 762–780. 32655169 10.1177/0013164417719308 PMC7328234

[41] M. L. Thomas , “Advances in Applications of Item Response Theory to Clinical Assessment,” Psychological Assessment 31, no. 12 (December 2019): 1442–1455. 30869966 10.1037/pas0000597 PMC6745011

[42] T. A. Slocum , S. E. Pinkelman , P. R. Joslyn , and B. Nichols , “Threats to Internal Validity in Multiple‐Baseline Design Variations,” Perspectives on Behavior Science 45 (2022): 619–638. 36249165 10.1007/s40614-022-00326-1 PMC9458807

[43] S. C. Ohiri , M. Momoh , O. Christopher , I. Ikeanumba , and C. Benedict , “Differential Item Functioning Detection Methods: An Overview,” International Journal of Research Publication and Reviews 5, no. 2 (February 2024): 1555–1564.

[44] D. Pisică , R. Dammers , E. Boersma , and V. Volovici , “Tenets of Good Practice in Regression Analysis. A Brief Tutorial,” World Neurosurgery 161 (May 2022): 230–239.e6. 35505539 10.1016/j.wneu.2022.02.112

[45] D. Thompson and B. Wesolowski , “Standard Error of Measurement,” in The SAGE Encyclopedia of Educational Research, Measurement, and Evaluation [Internet] (SAGE Publications, Inc, 2018), https://methods.sagepub.com/reference/the-sage-encyclopedia-of-educational-research-measurement-and-evaluation/i19746.xml.

[46] C. A. C. Prinsen , L. B. Mokkink , L. M. Bouter , et al., “COSMIN Guideline for Systematic Reviews of Patient‐Reported Outcome Measures,” Quality of Life Research 27, no. 5 (May 2018): 1147–1157. 29435801 10.1007/s11136-018-1798-3 PMC5891568

[47] A. Lluch , J. Kahn , A. Stricker‐Krongrad , O. Ziegler , P. Drouin , and L. Méjean , “Internal Validation of a French Version of the Dutch Eating Behaviour Questionnaire,” European Psychiatry 11 (October 1996): 198–203. 19698450 10.1016/0924-9338(96)88391-X

[48] J. Westenhoefer , A. J. Stunkard , and V. Pudel , “Validation of the Flexible and Rigid Control Dimensions of Dietary Restraint,” International Journal of Eating Disorders 26, no. 1 (1999): 53–64. 10349584 10.1002/(sici)1098-108x(199907)26:1<53::aid-eat7>3.0.co;2-n

[49] C. Framson , A. R. Kristal , J. M. Schenk , A. J. Littman , S. Zeliadt , and D. Benitez , “Development and Validation of the Mindful Eating Questionnaire,” Journal of the American Dietetic Association 109, no. 8 (2009): 1439–1444, 10.1016/j.jada.2009.05.006. 19631053 PMC2734460

[50] L. Hulbert‐Williams , R. Hastings , D. M. Owen , et al., “Exposure to Life Events as a Risk Factor for Psychological Problems in Adults With Intellectual Disabilities: A Longitudinal Design,” Journal of Intellectual Disability Research 58, no. 1 (2014): 48–60, 10.1111/jir.12050. 23627774

[51] T. L. Tylka and N. L. Wood‐Barcalow , “The Body Appreciation Scale‐2: Item Refinement and Psychometric Evaluation,” Body Image 12 (2015): 53–67. 10.1016/j.bodyim.2014.09.006. 25462882

