# Supplementary material for: Psychometric Properties of Eating Behaviour Instruments for Older Adults: A Systematic Review
Source: J Hum Nutr Diet. 2025 Sep 23;38(5):e70127. doi: 10.1111/jhn.70127 (PMC12457733; doi:10.1111/jhn.70127)
Supplement: Supplementary file 1 — SUPPLEMENTARY MATERIAL 1: Reasons discussed during the consensus meeting among researchers for the COSMIN classification presented in table 3. [file JHN-38-0-s001.docx]

| **SUPPLEMENTARY MATERIAL 1.** Reasons discussed during the consensus meeting among researchers for the COSMIN classification presented in Table 3 | | | | | | | | | |
| --- | --- | --- | --- | --- | --- | --- | --- | --- | --- |
| **Instruments/Author** | **Box 1.** | **Box 2.** | **Box 3.** | **Box 4.** | **Box 5.** | **Box 6.** | **Box 7.** | **Box 8.** | **Box  9.** |
| **1 - Three-Factor Eating Questionnaire-R18 (TFEQ -18)** | | | | | | | | | |
| Malkki-Keinänen  et al. (2022) | It is not clear whether there were group moderators or qualified interviewers. It was not reported whether a pilot study was conducted. | The approach used to analyze the target population’s responses to the instrument was not presented. | IRT/Rasch analysis was not performed. | IRT/Rasch analysis was not performed. | MGCFA, regression analysis, or DIF analysis were not performed. | The analysis was conducted, but the time interval between tests was not clearly stated. Testing conditions were not similar. ICC, Pearson, or Spearman correlation was not calculated. | Test conditions were not similar. | No other important  methodological  flaws. | Adequate description of most of the important  characteristics of the  subgroups. Assumable that statistical method  was appropriate. |
| **2- Modified Yale Food Addiction Scale Version 2.0 (MYFAS-2.0)** | | | | | | | | | |
| Pipová et al. (2020) | It is not clear whether there were group moderators or qualified interviewers. The number of researchers involved, and their respective responsibilities were not reported. It was also not mentioned whether a pilot study was conducted. | The approach used to analyze the target population’s responses to the instrument was not presented, and only one interviewer was reported for this stage. | IRT/Rasch analysis was not performed. | IRT/Rasch analysis was not performed. | MGCFA, regression analysis, or DIF analysis were not performed. | The analysis was conducted, but the time interval between tests was not clearly reported. ICC, Pearson, or Spearman correlation was not calculated. | SEM calculated based on Cronbach’s alpha. | Correlations were not calculated. | No other important methodological flaws |
| **3- The Dutch Eating Behaviour Questionnaire (DEBQ)** | | | | | | | | | |
| Bailly et al. (2012) | The pilot study was not conducted with a representative sample of the target population. The number of participants was inadequate for analysis. | No information is provided on how this stage was conducted, which analysis was performed, or how many interviewers were involved; however, the text describes the outcome as 'good'. Moreover, although the sample size is reported, it is insufficient. | IRT/Rasch analysis was not performed. | IRT/Rasch analysis was not performed. | The sample included fewer than five participants per item and fewer than 100 per group. MGCFA, regression analysis, or DIF analysis were not performed. | The analysis was conducted, but the time interval between tests and the testing conditions were not clearly reported. ICC, Pearson, or Spearman correlation was not calculated. | SEM calculated based on Cronbach’s alpha. | Correlations were not calculated. | Adequate description of the important characteristics of the subgroups and statistical method was appropriate |
| **4- Three-Factor Eating Questionnaire-51 (TFEQ-51)** | | | | | | | | | |
| Löffler et al.  (2015) | Inappropriate sample size for analysis. It was not reported whether a pilot study was conducted. | The approach used is explained, but the number of interviewers involved is not reported; however, the outcome is described as 'good' in the text. | IRT/Rasch analysis was not performed. | IRT/Rasch analysis was not performed. | MGCFA, regression analysis, or DIF analysis were not performed. | The analysis was conducted, but the time interval between tests was not clearly reported. ICC, Pearson, or Spearman correlation was not calculated. | SEM calculated based on Cronbach’s alpha. | Correlations were not calculated. | Adequate description of the important characteristics of the subgroups and statistical method was appropriate |
| **5- Modified Yale Food Addiction Scale (MYFAS)** | | | | | | | | | |
| Masheb et al. (2018) | It was not explicitly stated whether there were group moderators or qualified interviewers. | Insufficient sample size for analysis. | IRT/Rasch analysis was not performed. | IRT/Rasch analysis was not performed. | The sample included fewer than five participants per item but had at least 100 participants per group. MGCFA, regression analysis, or DIF analysis were not performed. | The analysis was conducted, but the time interval between tests was not clearly reported. ICC, Pearson, or Spearman correlation was not calculated. | SEM calculated based on Cronbach’s alpha | No other important  methodological  flaws. | Some information on measurement properties of the comparator instrument(s) in any study population |
| **6- Mindful Eating Behavior Scale (MEBS)** | | | | | | | | | |
| Winkens et al. (2017) | It was not explicitly stated whether there were group moderators or qualified interviewers. | No information is provided on how this stage was conducted, which type of analysis was performed, or how many interviewers were involved; yet, the text describes the outcome as 'good'. Additionally, although the sample size is reported, it is insufficient. | IRT/Rasch analysis was not performed. | IRT/Rasch analysis was not performed. | MGCFA, regression analysis, or DIF analysis were not performed | The analysis was conducted, but the time interval between tests was not clearly reported. ICC, Pearson, or Spearman correlation was not calculated. | SEM calculated based on Cronbach’s alpha. | No other important  methodological  flaws. | Adequate description of the important characteristics of the subgroups and statistical method was appropriate |
| **7- Food Avoidance Beliefs and Behaviors (FABB)** | | | | | | | | | |
| Yung et al. (2019) | It was not explicitly stated whether there were group moderators or qualified interviewers. | The approach used is explained, but the number of interviewers involved is not reported; nevertheless, the outcome is described as 'good'. | IRT/Rasch analysis was not performed. | IRT/Rasch analysis was not performed. | The sample included fewer than five participants per item but had at least 100 participants per group. MGCFA, regression analysis, or DIF analysis was not performed | No other important  methodological  flaws. | SEM calculated based on Cronbach’s alpha. | Correlations were not calculated. | Adequate description of the important characteristics of the subgroups and statistical method was appropriate |
| **8 - Mealtime Engagement Scale (MES)** | | | | | | | | | |
| Liu et al. (2021) | Inadequate sample size for analysis. No pilot study or cognitive interview was conducted. | The approach used is explained, but the number of interviewers involved is not reported; nevertheless, the outcome is described as 'good'. | IRT/Rasch analysis was not performed. | IRT/Rasch analysis was not performed. | The sample included fewer than five participants per item and fewer than 100 participants per group. MGCFA, regression analysis, or DIF analysis were not performed | It is assumed that participants were in a stable condition, but no explanation is provided on how this was determined. | SEM calculated based on Cronbach’s alpha. | No other important  methodological  flaws. | Poor of no description of the important characteristics of the subgroups |
| Note. NA – Not Applicable; VG – Very Good; A – Adequate; D – Doubtful; I – Inadequate; Box 1 - Instrument Development; Box 2 - Content Validity; Box 3 - Internal Validity; Box 4 - Internal Consistency; Box 5 - Cross-Cultural Validity / Measurement Invariance; Box 6 - Reliability; Box 7 - Measurement Errors; Box 8 - Criterion Validity; Box 9 - Hypothesis Testing for Construct Validity; MGCFA - Multiple-group confirmatory factor analysis; ICC - Intraclass Correlation Coefficient; DIF - Differential item functioning | | | | | | | | | |
